# Knowledge Gaps in Mobile Health Research for Promoting Physical Activity in Adults With Autism Spectrum Disorder

**DOI:** 10.3389/fpsyg.2021.635105

**Published:** 2021-03-24

**Authors:** Daehyoung Lee

**Affiliations:** Department of Applied Human Sciences, University of Minnesota Duluth, Duluth, MN, United States

**Keywords:** mHealth application, physical activity, adults with autism, behavior change techniques, gamification

## Abstract

A growing body of research highlights that adults with autism spectrum disorder (ASD) have poor health outcomes, yet effective health interventions are lacking for this population. While mobile health applications demonstrate potential for promoting physical activity (PA) in adults with ASD, scientific evidence for supporting this tool’s long-term effectiveness on PA behavior change remains inconclusive. This study aimed to provide the latest information on PA research and the prospective role of mobile health applications for promoting PA in adults with ASD. A literature review demonstrated that a few available studies show contradictory results regarding PA levels in adults with ASD, and behavior change techniques and gamification-guided mobile health applications can be promising tactics to leverage autism’s strengths and increase PA in these individuals. Optimizing design decisions based on needs analysis and user feedback is crucial to identifying and developing a sustainable mobile health intervention for PA promotion in adults with ASD.

## Introduction

Autism spectrum disorder (ASD) is a pervasive neurodevelopmental condition characterized by social interaction and communication deficits, as well as restricted or repetitive behavior patterns and interests (American Psychiatric Association, 2013). According to a recent report from the Centers for Disease Control and Prevention (CDC)’s Autism and Developmental Disabilities Monitoring Network in 2020, the estimated prevalence of ASD has continuously increased since its first surveillance in 2007. Currently, 1 in 54 children in the United States have been identified with ASD, a nearly 10% increase from a previous estimate of 1 in 59 children reported in 2018 (Baio et al., 2018; Maenner, 2020). A recent modeling study conducted by Dietz et al. (2020) provided the first surveillance data on the estimated prevalence of adults with ASD in the United States using multiple national data sets. The study estimated that approximately 2.21% of United States adults (18 years and older) were living with ASD in 2017, and the prevalence was higher in adult males than females, as consistent with previous findings on children with ASD (Baio et al., 2018; Kogan et al., 2018). As remarked in the study, however, research on autism in adulthood is still a challenge because psychometrically validated tests for screening adults with ASD are currently unavailable (Dietz et al., 2020). Additionally, those who live independently with typical cognitive function may not be accurately monitored due to a lack of connection with public or community settings (e.g., schools, therapeutic entities, etc.), as well as an absence of surveillance systems to address this issue (Dietz et al., 2020).

Despite the relatively little attention paid towards adults with ASD, research on risk factors for developing autism and understanding the condition’s impact on health outcomes has increased significantly over the past two decades (Wolff, 2004; Pellicano et al., 2014). Importantly, the existing literature has been consistent in showing that adults with ASD have poorer outcomes across health domains than those without ASD. Croen et al. (2015) studied a large sample of adults with ASD (*n* = 1,507) in the United States and highlighted that these individuals are at a higher risk for nearly all mental and physical health conditions (e.g., depression, anxiety, obesity, and diabetes) compared to adults without any ASD diagnoses. Furthermore, adults with ASD are more than twice as likely as their neurotypical counterparts to die prematurely (Hirvikoski et al., 2016). It is predicted that the shortage of resources and the existing health disparities for this population will likely expand (Tyler et al., 2011; Krahn et al., 2015). This descriptive mini review aimed to provide the latest information on physical activity (PA) research and the prospective role of mobile health applications for promoting PA in adults with ASD. Four electronic databases (Google Scholar, PubMed, Scopus, and Web of Science) were searched for studies published between October 2000 and September 2020. Search terms included “physical activity,” “mobile application,” “autism,” and a combination of these keywords and close variants (e.g., Asperger’s syndrome, mHealth, fitness app, etc.). The search was limited to articles published in English.

## PA in Adults with ASD

There is little information on PA as a preventive health behavior in adults with ASD (Reinders et al., 2019). Most research in this area has been conducted in children and adolescents, and the findings indicate that they are less physically active (Pan, 2008; Stanish et al., 2017) or engage in similar levels of PA participation as their typically developing peers (Sandt and Frey, 2005; Bandini et al., 2013). More importantly, moderate to vigorous PA (MVPA), which is the level of activity associated with health outcomes, significantly decreases with age in this population (MacDonald et al., 2011). Nonetheless, PA has been used with moderate success as a social or behavioral intervention in young individuals with ASD (Sowa and Meulenbroek, 2012; Schmitz Olin et al., 2017). A previous systemic review found that PA or exercise may be effective at reducing negative behaviors (e.g., aggression, elopement, and stereotypy) and increasing positive behaviors (e.g., academic responding, on-task behavior, and motor performance) in both children and young adults with ASD (Lang et al., 2010), although the relatively small sample (*n* = 64) and varying methodological quality in existing studies make it difficult to draw a solid conclusion about the effects of PA on behavioral outcomes in this population.

National and international health organizations recommend that adults should participate in at least 150 min of moderate-intensity PA or 75 min of vigorous-intensity PA throughout a week, or an equivalent combination of MVPA, to see health benefits (Piercy et al., 2018; World Health Organization, 2019). While the benefits of regular PA (e.g., improvement in muscular and cardiorespiratory fitness, reduced risk of cardiovascular diseases and mental disorders, and weight control) have been supported by numerous studies (Warburton et al., 2006; Warburton and Bredin, 2017), insufficient PA or physical inactivity is a leading risk factor for coronary heart disease and global premature mortality (Autenrieth et al., 2011; Lee et al., 2012). To date, there exist only a few available studies on PA in adults with ASD, and they have shown contradictory results (Eaves and Ho, 2008; Lalonde et al., 2014; Garcia-Pastor et al., 2019). Eaves and Ho (2008) used parent proxy reports and found that adults with ASD engaged in MVPA on average once per week and spent about 13 h/day sitting. Lalonde et al. (2014) were the first to use pedometers as an objective measure to promote walking activities in a small sample of adults with severe ASD. A walking program, along with goal setting, feedback on performance, and reinforcement, was effective at increasing daily steps to a desired level (e.g., >10,000 steps/day) in all participants. As a pilot surveillance study, Garcia-Pastor et al. (2019) used uniaxial accelerometry and found that Spanish adults with ASD met the recommended PA guidelines of ≥150 min of MVPA a week, but they were also highly sedentary. It is important to note that, however, sample characteristics in these studies were described as having moderate to severe autism symptoms and relatively low levels of cognitive function, needing high levels of support. Most of the study participants attended segregated schools or lived with parents, in supported group homes, or foster care. Moreover, the methodological heterogeneity in PA assessment (i.e., proxy survey reports vs. objective measures) makes it difficult to synthesize previous findings. As such, it is inconclusive how PA is associated with varying levels of cognitive function and the concordance between subjective and objective measures for PA assessment in adults with ASD.

## Mobile Health Applications For People with ASD

Mobile application is an attractive modality for behavioral intervention in people with ASD because human-technology interaction enables visuospatial learning, which is often noted as a particular strength in those with ASD (Samson et al., 2012). It also presents a lower social burden compared to traditional face-to-face interaction, an area in which those with ASD struggle, as technology provides a predictable and consistent social platform (Kientz et al., 2013). With the increased accessibility and affordability, the use of mobile applications for people with ASD has been widespread across a range of domains focusing on visual cues as a supplementary intervention. Autism-friendly mobile applications are particularly designed to accommodate the unique characteristics of autistic users (e.g., avoidance of social situations and strengths in logical reasoning; American Psychiatric Association, 2013; Shah et al., 2016). A number of user-centered educational applications have gained popularity in the autism community by providing leaning opportunities for social and communication skills, emotional regulation skills, and occupational functioning (e.g., appropriate behavior in the workplace or school and problem-solving skills; Ayres et al., 2013; Hourcade et al., 2013; Gay and Leijdekkers, 2014).

However, there exist almost no similar attempts to use mobile applications to address PA disparities in individuals with ASD. The only related effort has been a video modeling mobile application, Exercise Buddy, that was developed to help children with ASD acquire and improve fundamental motor skills consisting of locomotor (e.g., walking, running, and jumping), balance, and object control skills (Bittner et al., 2017). With supportive visual and auditory instructions, this video modeling program effectively increased heart rate and energy expenditure in children with ASD while they performed locomotor skill tasks (Bittner et al., 2017). However, there was no *post hoc* investigation on the functional outcomes in relation to actual motor skill acquisition/improvement after the intervention and any consequent impact on PA or other daily living activities in real-world settings. In addition, the system focused on children with limited cognitive function and required substantial assistance for proper execution; thus, it cannot be applicable to adults with ASD who live independently and make their own choices in their health behaviors.

Although the evidence supporting their long-term effectiveness on health behavior change is unclear, mobile health applications can have small to moderate effects in the short term (e.g., up to 3 months) on self-monitoring or changing PA and sedentary behavior in overweight and sedentary neurotypical adults (Turner-McGrievy et al., 2013; Romeo et al., 2019). PA in adults with ASD may be best stimulated by using gamified mobile applications, particularly considering reports that adults with ASD use technology devices over 4 h/day, primarily playing games (Lee et al., 2018). Gamified behavioral interventions have rapidly expanded the technological potential to monitor and improve PA participation in a playful way (Zuckerman and Gal-Oz, 2014; Alahäivälä and Oinas-Kukkonen, 2016), which leverages the merits of a heightened sense of autonomy, as well as feelings of fun and continuation desire (Deterding et al., 2011; King et al., 2013). Also, an emerging body of research suggests that behavior change techniques (BCTs)-guided mobile health interventions can be useful to increase PA in inactive or sedentary individuals (Direito et al., 2017; Sullivan and Lachman, 2017). The importance of incorporating evidence-based BCTs should be amplified when designing health behavior change interventions for those with ASD, considering their restricted repertoires of behavior patterns and low social motivation (Dawson et al., 2005; Chevallier et al., 2012).

## BCTs and Gamification For PA Promotion in Adults with ASD

BCTs are a systematic, evidence-based health behavior change strategy for altering or redirecting causal processes that regulate healthy behaviors (Abraham and Michie, 2008; Michie et al., 2013). For example, some intervention strategies can be designed to monitor and promote PA participation, smoking abstinence, a healthy diet, or consistent sleep patterns by augmenting facilitators and/or mitigating barriers for behavior change (Carey et al., 2019). Since Abraham and Michie (2008) structured the first cross-behavior taxonomy of 22 BCTs, this strategy has been extensively adopted across the globe to design, implement, and evaluate behavior change interventions. On the basis of a systematic review and refinement, a current taxonomy of the BCTs includes 93 hierarchically clustered techniques, including self-monitoring of behavior, social support, goal setting, feedback on healthy behavior, action planning, incentives, etc., that offer multidisciplinary acceptance and use for developing effective behavior change interventions (Michie et al., 2013).

Effective behavior change interventions generally leverage multiple BCTs to implement health behavior change, and a mobile application enables researchers to deliver diverse BCTs in a time‐ and cost-effective way in real-world settings (Bird et al., 2013; Middelweerd et al., 2014; Crane et al., 2015). Currently, there are more than 325,000 health applications specifically designed for promoting physical fitness and PA in major application stores such as Google Play and the Apple App Store (Research2Guidance, 2017). Some of the available applications have empirically demonstrated the potential to enhance users’ PA engagement, but only a minimal number of the applications have been developed or evaluated through scientific methods, while most are not guided by evidence-based health intervention strategies such as BCTs (Cowan et al., 2013; Pagoto and Bennett, 2013).

Conroy et al. (2014) examined 167 top-ranked PA-promoting applications on the commercial market to verify the prevalence of BCTs among the popular PA applications. Specifically, they characterized the BCTs by analyzing the applications’ online descriptions and functions on the basis of the BCTs taxonomy. Among others, provision of instruction for behavior performance, demonstration of behavior, provision of contingent feedback on performance, behavioral goal-setting, and plans of social support or change were the most commonly adopted BCTs in the included applications (Conroy et al., 2014; Yang et al., 2015). Importantly, the majority of contemporary PA-promoting applications lacked BCTs as a theoretical background for the developmental process (Conroy et al., 2014). Due to the scarcity of systematic assessment within the contemporary PA applications, it is unclear how the incorporated BCTs affected and changed users’ health behavior, as well as how any positive health outcomes after the application use were associated with varying intrinsic or extrinsic motivation factors (Sullivan and Lachman, 2017).

Despite the limitations, gamified behavior change interventions in particular have presented promising results to promote PA in adults with and without ASD. Gamified interventions leverage game-like elements such as leaderboards, animated avatars, problem-solving, and attractive storylines to elevate the level of motivation and user engagement (Alsawaier, 2018). Chen and Pu (2014) deployed “HealthyTogether,” an interactive mobile fitness application, which focused on gamified social interaction among users leveraging social incentives and a point reward system. The research team found a significant relationship between PA and the number of messages that participants exchanged, implying the importance of cooperative social interaction in gamified PA contexts. The success of Pokémon Go, an augmented reality-based application that links a Pokémon character hunting game to location tracking technology, further heightened the emerging potential of using gamification techniques to promote achievable PA (e.g., walking) in real-life settings, even though the intervention effect was short-term (Althoff et al., 2016; Howe et al., 2016). Furthermore, Lee (2020) reported that “PuzzleWalk” a gamified, BCTs-based mobile application, can be effective at increasing user engagement and potentially PA in adults with high-functioning ASD. Their preliminary deployment study compared PuzzleWalk to a commercially popular PA-tracking application, Google Fit, to evaluate the efficacy of both apps on increasing PA in adults with ASD who do not have intellectual disability, but struggle with daily living skills using a randomized controlled trial design (*n* = 24). Findings indicated that PuzzleWalk users spent significantly larger amounts of time on application use compared to Google Fit users throughout a 5-week intervention period. Even though statistical significance was not reached, PuzzleWalk also demonstrated the potential for increasing MVPA in the short term and reducing sedentary time in adults with high-functioning ASD (Lee, 2020).

Taken together, a growing body of research supports the potential of gamification and BCTs-guided mobile applications for promoting PA in diverse population groups, including adults with ASD. However, despite the increasing popularity of using these promising tactics in commercial PA applications, evidence-based mobile health interventions are lacking, and only a few attempts have been made to address the unique characteristics and health needs in adults with ASD. Furthermore, more research is warranted to better understand the impact of varying degrees of cognitive abilities and sociability on health behaviors in adults with ASD.

## Concluding Remarks

This brief review has revealed that there is a critical need for interventional studies that leverage autism’s strengths to address preventive health and reduce health disparities in adults with ASD. Despite the seemingly successful expansion of mobile health applications for promoting PA in both adults with and without ASD, most of the existing health and PA applications on the commercial market are not designed using scientific evidence or health behavior theories such as BCTs (Middelweerd et al., 2014; Yang et al., 2015). It is also crucial to note that reports on the success of gamified applications are largely based on anecdotal evidence, and there is insufficient scientific evidence to support their long-term effectiveness in inducing voluntary behavior change, including PA engagement (King et al., 2013; Lister et al., 2014). The use of gamification principles in most popular PA applications does not adhere to any industry-standard or professional guidelines (Lister et al., 2014). Although BCTs and gamification are promising tactics for increasing user interest and PA motivation, it is inconclusive whether these features can lead to sustained behavior change. Furthermore, due to an absence of versatility in addressing the unique behavioral and social preferences of individuals with ASD (e.g., adherence to routines and visual preference for social stimuli; Leekam et al., 2011; Crawford et al., 2016), general mobile health applications are less likely to enact a successful PA behavior change in adults with ASD who tend to have relatively low self-esteem and intrinsic motivation for health behavior change (Koegel and Mentis, 1985; Cooper et al., 2017). To develop a sustainable mobile health intervention that promotes PA in adults with ASD, it is critical to optimize design decisions based on an in-depth understanding of the target users and iterative inquiry regarding user feedback and behavior patterns (i.e., user-centered design) to facilitate the use of and adherence to interventions (Mummah et al., 2016; Lee et al., 2020).

## Author Contributions

The author confirms being the sole contributor of this work and has approved it for publication.

### Conflict of Interest

The author declares that the research was conducted in the absence of any commercial or financial relationships that could be construed as a potential conflict of interest.
